# Recurrent & non-recurrent copy number variants in Native Americans and a cosmopolitan sample in relation to Alcohol Use Disorder and other psychiatric diseases

**DOI:** 10.21203/rs.3.rs-8108448/v1

**Published:** 2025-12-11

**Authors:** Salma M Wakil, Keita Morisaki, Pei-Hong Shen, Dylan G Sucich, Melanie Schwandt, Fielding Hejtmancik, Cheryl Marietta, Qiaoping Yuan, Nancy Diazgranados, Colin A Hodgkinson, David Goldman

**Affiliations:** National Institute on Alcohol Abuse and Alcoholism; National Institute on Alcohol Abuse and Alcoholism; National Institute on Alcohol Abuse and Alcoholism; National Institute on Alcohol Abuse and Alcoholism; National Institute on Alcohol Abuse and Alcoholism; National Eye Institute; National Institute on Alcohol Abuse and Alcoholism; National Institute on Alcohol Abuse and Alcoholism; National Institute on Alcohol Abuse and Alcoholism; National Institute on Alcohol Abuse and Alcoholism; National Institute on Alcohol Abuse and Alcoholism

**Keywords:** Copy number variation, Alcohol Use Disorder, Substance Use Disorder, Psychiatric Disease, Velocardiofacial Syndrome

## Abstract

**Background:**

Copy Number Variants (CNVs) can alter disease susceptibility by gene deletion, duplication and other mechanisms. CNVs are implicated in neuropsychiatric diseases. However, their rarity or *de novo* nature impedes linkage analysis. Therefore, we identified recurrent CNVs (rCNVs) in Native Americans with low genetic admixture and high prevalence of Alcohol Use Disorder (AUD) and other psychiatric disorders.

**Results:**

Large (> 200 kb) rCNVs were abundant in PI and SWI, almost all carrying at least one rCNV, and with some CNVs found in both geographically and linguistically distinct tribes. In patients carrying rCNVs, gene deletions led to haploinsufficiency, and duplications overexpression. Haplotype analysis revealed a common chromosome 6p21.33 rCNV that persisted in Native Americans for at least 750 generations, leading to haploinsufficiency of at least two genes. Gene-based CNV burden did not predict AUD or other psychiatric disorders. However, an rCNV, found in PI and duplicating three genes within the 22q11.2 Velocardiofacial Syndrome region, may be associated with psychiatric disease. Among 27 heterozygotes, 22 had AUD (OR = 3.18 [1.18–8.59], p = 0.01), and 24 had a psychiatric diagnosis (OR = 4.8 [1.4–16], p = 0.006, FDR 0.07 adjusted for 13 common rCNVs tested).

**Conclusion:**

Recurrent CNVs are prevalent in Native American populations and have ancient origins. While gene-based CNV burden did not predict AUD or other psychiatric disorders, specific rCNVs, such as those within 22q11.2 region, may confer higher risk for psychiatric conditions. Other less abundant rCNVs and non-recurrent CNVs might also alter risk, the effects of such CNVs being undetectable via genome-wide association studies with single SNPs.

## Background

Copy Number Variations (CNVs) are one of the most phenotypically significant classes of genomic variation. These structural variants range in size from indels of single nucleotides to indels of large chromosomal regions. CNVs are far less common than single nucleotide polymorphisms (SNPs) that number approximately 22 million (1) or rarer single nucleotide variants (SNVs) that are even more numerous. However, because of their larger size, CNVs contribute a comparable amount of nucleotide heterozygosity. On average, individuals may carry a burden of 1–2 deleted or duplicated genes attributable to large CNVs (2). CNVs intersecting gene regions can thus modify disease susceptibility by altering gene expression, or in a more nuanced fashion by structurally altering transcripts or post-transcriptional aspects of gene regulation (3). CNVs involving large chromosomal segments that duplicate or delete genes are thought to be subject to strong negative selection and to therefore be rare and persistent for only a few generations. Many are only observed as *de novo*, sporadic mutations. For example, Velocardiofacial Syndrome (VCF), caused by large, multigene, deletions at 22q11.2 occurs *de novo* in 90% of cases (4, 5). Structural variation due to different mutational origins contributes to variable expressivity and penetrance.

Large CNVs have been robustly associated with diverse disorders including autism, schizophrenia, type I diabetes, congenital abnormalities and neurodegenerative diseases (6–10). These CNV-phenotype associations have been discovered for CNVs that are individually rare and of different mutational origins, but that tend to recur in regions that represent recombination hotspots, sites of nonallelic homologous recombination (NAHR) or locations of L1 retro transposition (11, 12).

The psychiatric disease burden attributable to CNVs is unknown but may be substantial. In total and including 22q11.2 deletions, CNVs contribute as much as 10% of the genetic risk of schizophrenia (2, 13–15). The CNVs contributing to schizophrenia are primarily non-recurrent, occurring at the same chromosomal regions, and representing separate, or homoplasic, mutational events. Similarly, numerous CNVs have been implicated in developmental delay, intellectual disability (ID) and autism, contributing an estimated 14% of the genetic risk to these interrelated disorders (16–18).

The role of CNVs has been investigated in other neuropsychiatric disorders but is still largely unknown. Recently, the ability of CNVs to alter brain function and an approach to systematically screening for functional consequences of CNVs was demonstrated using single cell spatial transcriptomics (19). However, psychiatric disorders are multifactorial, and their vulnerability can be influenced by many loci of small molecular and downstream phenotypic effects, as shown by GWAS. A meta-analysis found significant enrichment of smaller CNVs located primarily in intergenic regions and enhancers in individuals with Major Depressive Disorder (MDD), suggesting a role for CNVs in altering gene expression and increasing MDD risk (20). In the large UK Biobank sample, known neurodevelopmental CNVs conferred a 1.25–1.3 OR (odds ratio) for depression, with no effect detected for other CNVs, perhaps because of either the rarity of these CNVs or their effect sizes. CNVs at 1q21.1, 16p11.2 and the 15q11–13 Prader-Willi region were associated with self-reported depression. Thus, the CNVs implicated in depression seem to be pleiotropic, conferring risk to other phenotypes in addition to depression (21–25). In a smaller sample of children, large CNVs were associated with anxiety and depression (26). Children with ADHD were more likely to carry *de novo* CNVs (27).

The relationship of CNVs to Alcohol Use Disorder (AUD) and other Substance Use Disorders (SUDs) is unknown. Remarkably, in a large, epidemiologically representative sample (NESARC III), DSM-5 AUD affected 29.1% of the U.S. population on a lifetime basis (28) and the one-year prevalence of AUD was estimated to be 13.9% (www.niaaa.nih.gov). Unlike other psychiatric disorders that may be triggered by exposures, AUD and other SUDs are dependent on a chosen exposure. Nevertheless, these disorders are moderately to highly heritable, and cross-transmitted, apparently because of shared genetic influences on processes such as reward, executive cognition and negative emotion, that are common to the vulnerability and progression of different addictive disorders, as well as other psychiatric disorders (29). As is true for other psychiatric disorders, many genes of small effect contribute to AUD and other SUDs, as demonstrated by GWAS. Furthermore, the advent of polygenic scores has confirmed that SUDs are cross transmitted with each other and other phenotypes as well (30, 31).

Regarding the contribution of specific CNVs to AUD, an association study linked CNVs at the16q12.2 and 9p21.2 regions to AUD. However, these effects, although large in magnitude, were not replicated in other studies that instead implicated CNVs in other regions (32). Notably, the number of people carrying a CNV at any region was always small, and furthermore those CNVs were molecularly distinct in different individuals. The observed role of CNVs in schizophrenia and autism suggests that it would be valuable to study large numbers of cases and controls carrying CNVs affecting the same region, and if possible, carrying the identical, recurrent CNV (rCNV). We performed these studies in two Native American Indian tribes with high rates of AUD and other psychiatric disorders, comparing the effects of rCNVs and overall CNV burden to a cosmopolitan sample of AUD cases and controls collected at the NIH Clinical Center (NIH CC).

## Results

**Figure 1:**
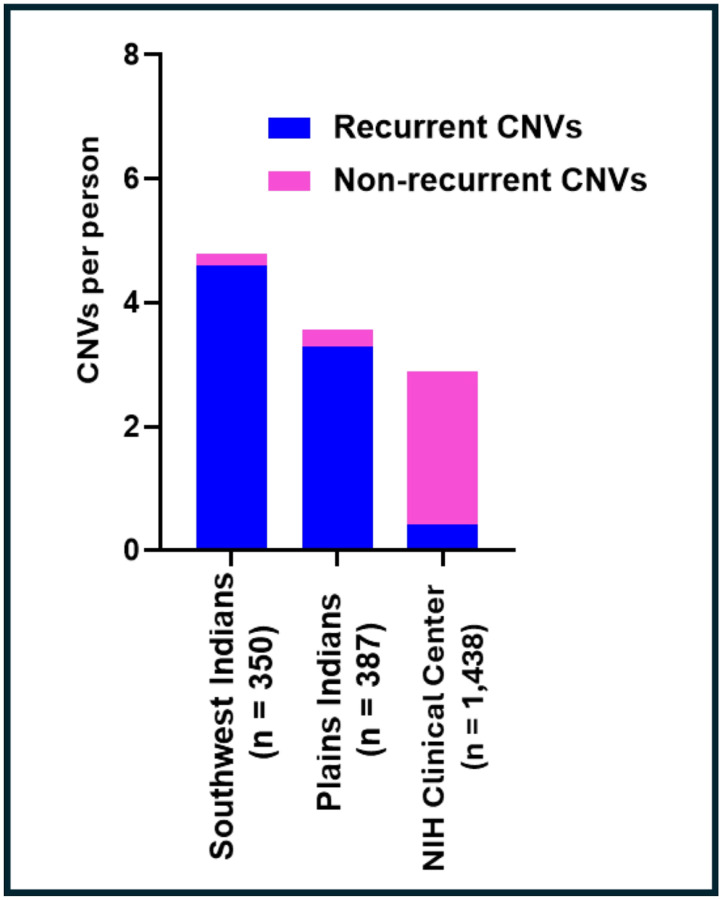
Nonrecurrent (rare) and recurrent CNVs (rCNVs) in three psychiatrically characterized samples The average number of recurrent (blue) and non-recurrent (pink) CNVs per individual are shown. CNVs were identified from SNP array data and classified as recurrent if observed in multiple individuals while nonrecurrent CNVs were unique to single individuals.

**Figure 2:**
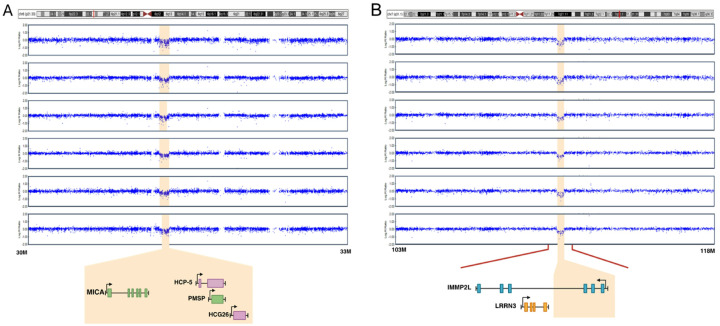
Signal intensities of two rCNVs causing deletions The 6p21.33 (A) and 7q31.1 (B) rCNVs identified in 154 and 22 individuals from two Native American populations led to decreased Log R Ratio (LRR) of SNPs within the respective regions. Genes within the 98 kb deletion of 6p21.33 were *MICA, HCP5, PMSPand HCG26*, and within the 156 kb region of 7q31.1, *IMMP2L* was partly deleted.

**Figure 3:**
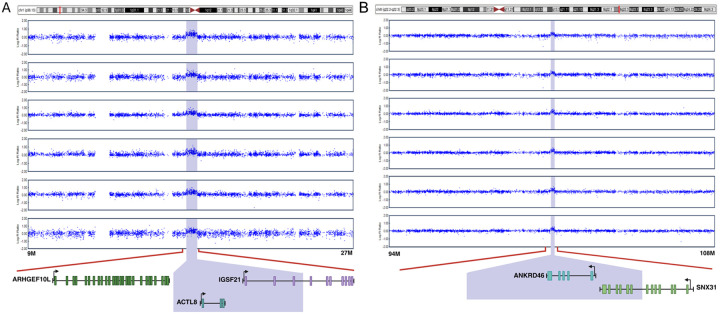
Signal intensities of two rCNVs causing duplications The 1p36.13 (A) and 8q22.2-q22.3 (B) rCNVs identified in eight and six individuals from two Native American populations led to increased Log R Ratio (LRR) of SNPs within the respective regions. Within the 623 kb deletion of 1p36.13, *ACTL8* was duplicated and *IFSF21was* partly duplicated. Within the 190 kb duplication, *ANKRD46* was duplicated and *SNX31* was partly duplicated.

Coincidentally, the Plains Indians (PI), Southwest American Indian (SWI) and NIH CC samples had similar ratios of AUD cases to controls, the fractions of AUD cases being 0.60, 0.71 and 0.62, respectively (**Table S1**). These high prevalences reflect the abundance of AUD in the PI and SWI communities, and the ascertainment bias of the NIH CC sample for research on AUD. Additionally, these cohorts had high prevalences of other psychiatric disorders (Tables 1 & **S1**). The overlap between CNVs detected by both CNV Partition and Penn CNV, and that were used in subsequent analyses, was high (0.956). There were a total of 1384 CNV events in PI, 1696 in SWI and 4179 in the NIH CC sample, translating to an average of 3.6 CNVs per person in PI, 4.8 in SWI and 2.9 in the NIH CC sample (Figure 1). Of these CNV events, 939 (68%), 1054 (62%), and 1143 (27%) were deletions and reciprocally 445 (32%), 642 (38%) 3036 (73%) were duplications in PI, SWI, and the NIH CC sample, respectively (Figures 2–3). Thus, deletions were more common in the Native Americans (p ≪ 0.001). The average size of all CNVs (including both deletions and duplications) was 317 kb in PI, 192 kb in SWI, and 269 kb in the NIH CC sample.

**Figure 4:**
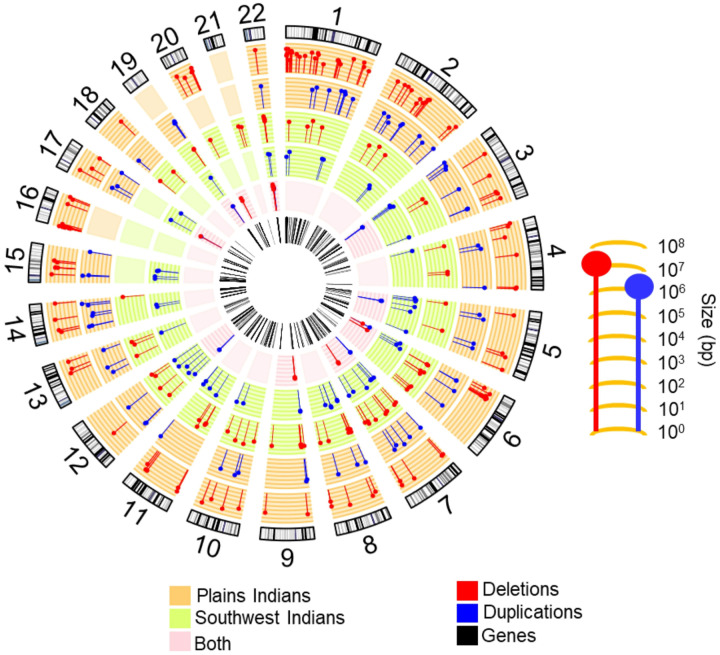
rCNVs are distributed across the genome in two Native American tribes Circos tracks are as follows (from outer to inner): (1) Locations of rCNVs on the GRch37 karyotype: deletions (red) and duplications (blue); (2,3) Orange tracks- rCNVs observed in Plains Indians, the length of symbols (**j**) corresponding to sizes, ranging up to 10 Mb (10^7^ bp), as shown; (4,5) Green tracks- rCNVs observed in Southwest Indians, the lengths of symbols (**j**) again corresponding to sizes; (6) Pink track- rCNV deletions (red) and duplications (blue) common to both Native American populations; and (7) the bars in the innermost track correspond to genes deleted, duplicated or partially deleted or duplicated by rCNVs.

Compared to the NIH CC sample, Native Americans had proportionately fewer unique (non-recurrent) CNVs but harbored far more recurrent CNVs (rCNVs) (Figure 4). In Native Americans, several rCNVs were observed in more than 45 individuals, and sixteen rCNVs were found in both Native American tribes (Table 2). We tested whether the overall higher frequency of rCNVs observed in SWI was related to fact that this tribe has undergone less admixture, correlating level of admixture with the number of rCNVs that individuals carried, however, there was no significant correlation in either population (**Figure S3**). The absence of relationship of abundance of rCNVs to admixture was also borne out at the level of individual rCNVs, wherein some rCNVs were more frequent in SWI, others more frequent in PI and some rCNVs were observed in only one population. Overall, admixture of both Native American populations with non-Native Americans was low.

**Figure 5:**
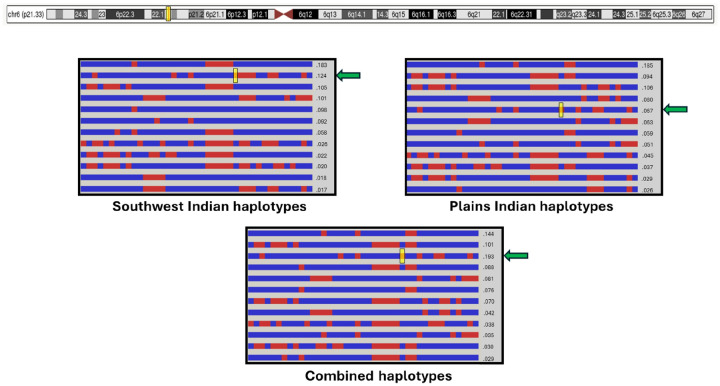
Distinct haplotypes shared by 6p21.33 rCNV chromosomes in both Southwest Indians and Plains Indians This figure shows distinct shared haplotypes in the region of the 6p21.33 rCNV. The yellow boxes denote the location of the 6p21.33 deletion, and the green arrows show the haplotypes containing the 6p21.33 rCNV. The frequency of the CNV haplotype (and CNV) was 0.121 in Southwest Indians (N=350) and 0.077 in Plains Indians (N=387).

We observed that each rCNV occurred on a characteristic haplotype (Figure 5) thus confirming their identity as recurrent, rather than being CNVs that happened to recur in the same chromosomal region and to share the same boundaries. This consistency of haplotype background included rCNVs common to both Native American populations, although the genotyping arrays were different, and therefore the exact SNPs constituting the conserved haplotypes differed between PI and SWI.

**Figure 6:**
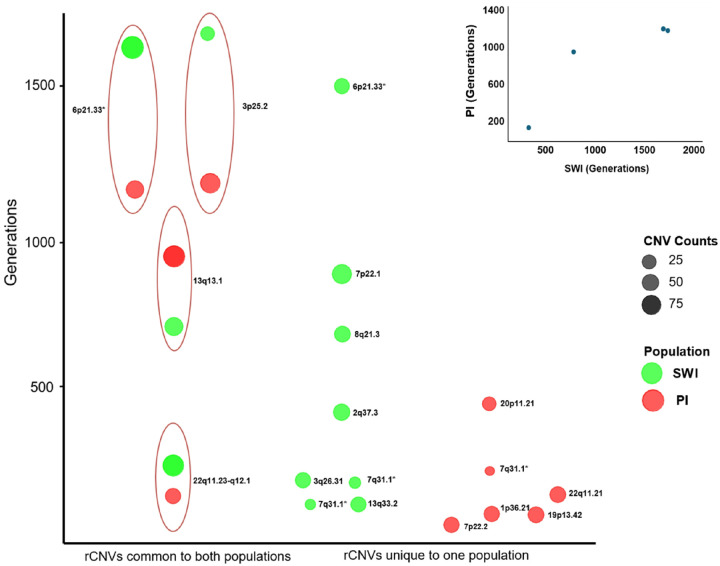
Coalescence times, in generations, of rCNVs in one or both Native American populations Shown are four rCNVs present in both SWI and PI (left), eight rCNVs detected only in SWI (center), and six rCNVs detected only in PI (right). Coalescence times were computed using the Gamma method based on the first recombination event observed 5’ and 3’ to the rCNV and using local average recombination rates. As shown in the inset, coalescence times for the four rCNVs identified in both Native American populations tended to correlate although the p-value was not computed because N was <5. (* denotes different rCNVs at 7q31.1).

The consistency of haplotypes backgrounds, along with common breakpoints, strongly suggested that each rCNV derived from a common ancestor, even the rCNVs observed in both geographically separated and linguistically distinct Native American populations. The haplotype coalescence times of the rCNVs were thereby calculated via the Gamma method based on sizes of the conserved haplotypes on which the rCNVs resided, and by using the recombination rate local to the chromosomal region (Figure 6). The rCNVs observed in both Native American populations were ancient, apparently present in ancestors of the PI and SWI for hundreds of generations to more than a thousand generations, as required for recombinants to be likely to arise within the 5’ and 3’ flanking haplotypes close to the CNVs themselves. The difference in ages of rCNVs observed in both Native American populations versus those observed in only one was statistically significant (Mann-Whitney U test, p = 0.01). Several of the rCNVs observed in both Native American populations appeared to have arisen > 10,000 years ago (Figure 6), representing either *de novo* mutations in early Native Americans or origins in a common Asian ancestor. The rCNVs that were more abundant tended to be more ancient (r = 0.445, slope = 0.0194, p < 0.05).

In locations, the rCNVs were broadly distributed across the autosomes (Figure 4). Overall, 75% of rCNVs identified in Native Americans contained RefSeq genes, suggesting that many of these rCNVs may directly alter gene expression. Additionally, many of the non-gene containing rCNVs were still proximal to genes. The genes deleted, duplicated or potentially impacted by the rCNVs are listed in Table 2 with more detailed information provided in Tables S2, S3 and S4. It will be shown that rCNVs were inherited in Mendelian fashion within kindreds yet pervasive throughout the populations, with carriers of any particular rCNV not having notably higher kinship coefficients compared to noncarriers.

### Functionality of the 6p21.33 rCNV, and other rCNVs

We evaluated the functionality of the 96kb deletion at 6p21.33 (allele frequency 0.077 in PI and 0.121 in SWI) by transcriptome analysis of five PI lymphoblastoid cell lines (LCLs) heterozygous for this rCNV versus four noncarriers. This analysis also enabled us to test, on a more limited basis, the functionality of other rCNVs and CNVs that were heterozygous in the nine LCLs, and in instances where genes duplicated or deleted by the CNVs were ordinarily expressed in LCLs. We evaluated both *cis* and *trans* consequences of the 6p21.33 rCNV for mRNA expression. Two of the genes within the region deleted by the 6p21.33 rCNV, *MICA* and *HCG-26*, were expressed at measurable levels, and their expression was reduced by approximately 50% in individuals heterozygous for the deletion (Figure S4). Consistent with other reports, expression of the two other genes within the deleted region, *HCP-5* and PMSP, was not detected. The uncompensated reduction of *MICA* and/or *HCG-26* expression appears to have led to extensive *trans* effects on the expression of other genes. Differential gene expression analysis identified 473 genes (|FC| > 1.25; nominal p-value < 0.05), of which 202 were upregulated, and 271 were downregulated, and among these 34 remained significant after FDR correction (**Figure S5**). These differentially expressed genes implicated several canonical pathways, including Molecular Mechanisms of Cancer, RHO GTPase cycle, Hepatic Fibrosis Signaling, Serotonin receptor Signaling and G-Protein Coupled receptor Signaling.

The expression levels of ten additional genes were potentially impacted in direct (*cis*) fashion by heterozygous duplications or deletions in these nine LCL lines. Each of these additional rCNVs was represented in only one or two heterozygous cell lines. Therefore, to test for *cis* effects on expression we normalized levels of expression of genes these rCNVs contained to the non-CNV homozygote and thereby performed a combined analysis of the *cis* effects of the rCNVs on gene expression. As shown in Figure S6, heterozygous deletions reduced expression of genes by approximately 50%, whereas duplications increased expression by approximately 50%.

### Associations of individual rCNVs

In this study, there was insufficient power to detect small risk effects for psychiatric disorders, even for rCNVs. However, to detect large effects of rCNVs on AUD and “Any Psychiatric Disorder”, we tested individual rCNVs observed in ≥25 carriers in PI (10 rCNVs), or ≥ 22 carriers in SWI (12 rCNVs), with three rCNVs shared across both populations. An rCNV duplication at 22q11.21, observed only in PI, was most robustly associated, being enriched both in AUD cases (OR = 3.18 [1.18–8.59], p = 0.01) and “Any psychiatric disorder” (OR = 4.77 [1.41–16.1], p = 0.006). Although both p-values were below 0.05, neither remained statistically significant after Bonferroni correction for testing of 13 rCNVs carried by ≥25 PI. Other CNVs also showed nominally elevated ORs without reaching statistical significance; deletions at 19q13.42 were enriched in individuals with AUD (OR = 1.75 [0.75–4.09], p = 0.09) and with “Any psychiatric disorder” (OR = 2.01 [0.79–5.11], p = 0.13). Similarly, deletions at 7p22.2 showed increased odds for AUD (OR = 2.02 [0.83–4.90], p = 0.11) and for “Any psychiatric disorder” (OR = 1.62 [0.67–3.94], p = 0.28). In addition, deletions at 8p23.2 were modestly enriched among cases with AUD (OR = 1.80 [0.73–4.42], p = 0.19) and for “Any psychiatric disorder” (OR = 1.80 [0.70–4.63], p = 0.21). Among SWI, some of the 12 tested rCNVs such as deletions at 2q37.3 (OR = 2.12 [0.85–5.29], p = 0.10) and 7p36.1 (OR = 1.57 [0.57–4.37] p = 0.38) exhibited elevated ORs for AUD with wide confidence intervals, suggesting potential associations that may require larger sample sizes for validation. Notably, none of the three rCNVs observed in both populations were nominally associated with either AUD or “Any psychiatric disorder” (Table 3). In addition, deletions at 20p12.1 in the PI (n =16 carriers) showed strong association with AUD (OR = 4.96 [1.11–22.1], p = 0.02) and a suggestive association with “Any psychiatric disorder” (OR = 4.02 [0.90–17.9], p = 0.049). While these associations did not remain significant after Bonferroni correction, the elevated ORs may deserve future attention.

### CNV burden and psychiatric disease

To evaluate consequences of CNV burden, we combined the PI and SWI samples, and also examined the effect of CNV burden in the NIH CC sample (**Table S1**). Comparing the rank order of cases and controls, there was no overall significant effect of CNV burden on either AUD or “any psychiatric diagnosis”. The CNV gene load was numerically higher in AUD cases when PI and SWI datasets were combined (mean ± SD: 15.27 ± 59.2) compared to non-AUD controls (13.36 ± 50.17), but the difference was not statistically significant. In secondary analyses, for Post-Traumatic Stress Disorder (PTSD), CNV burden was higher whether measured by number of CNVs (6.51± 6.93 vs. 4.02 ± 3.28) or gene load (31.41 ± 86.37 vs. 13.16 ± 52.64). However, this difference did not survive correction for multiple testing.

## Discussion

CNVs deleting or duplicating genes are not rare. In this study, among 387 PI, 350 SWI, and 1438 individuals from the NIH Clinical Center we identified only 145 individuals out of 2175 (12, 10 and 123 respectively), in whom a CNV did not alter the copy number of at least one gene. These large CNVs deleting and duplicating genes are likely to be consequential and as recently has been explored for several CNVs via transcriptome analysis of postmortem brain (19). Here, we showed that the haploinsufficiency caused by deletion or gene excess due to duplication was not compensated for a 6p21.33 rCNV in the MHC region nor for a series of other rCNVs harboring genes expressed in LCLs. For the 6p21.33 rCNV, the one rCNV we tested using multiple cell lines stratified to be heterozygotes or noncarriers for the rCNV, we also observed a cascade of *trans* effects. Indeed, dosage compensation is thought to be the exception, rather than the rule, in eukaryotes, for example genes deleted in *Saccharomyces* (33) or duplicated in humans via Trisomy 21.

The importance of the lack of dosage compensation is amplified by the fact that many of the genes deleted or duplicated in the rCNVs have been implicated in heritable diseases (**Table S2**). Potentially, large CNVs could contribute to the so-called missing heritability of psychiatric diseases and other phenotypes, and furthermore *de novo* CNVs represent a genetic origin of variance that, in many or even most instances, would not contribute to heritability. A common deletion of the *GSTM1* (Glutathione s-transferase) gene reduces enzymatic activity and heightens oxidative stress, especially in combination with xenobiotics such as are found in tobacco smoke. The *GSTM1*-null genotype promotes cancers, metabolic and autoimmune disorders, but absence of GSTM1 activity can itself be partially compensated by the overexpression of other GST family members (34). Often, linkage of CNVs to phenotypes is impeded by their low frequencies, and divergences in molecular effects arising from differences in CNV breakpoints.

The rCNVs we identified in two Native American Indian tribes may be recurrent due to founder effects, population bottlenecks and/or relatively small effective breeding sizes, these representing interrelated but somewhat distinct possibilities. Indeed, both of these Native American populations have experienced a reduction in STR (Short Tandem Repeat) diversity, heterozygosity across STR loci being reduced from approximately 0.7 to 0.6, compared to cosmopolitan, non-African populations (35). Alternatively, the rCNVs could be maintained by balanced selection, but this is less likely as it would not explain why rCNVs are rare in the NIH CC sample and other cosmopolitan populations.

Both population isolates and families represent sampling frameworks in which private alleles are more likely to be observed, (36) and studies of genetic diseases in these contexts has led to many discoveries in medical genetics. The rCNVs we observed in Native Americans are not private mutations in the sense that they are not limited to individual families, or even a single tribe. Instead, the rCNVs were widely distributed in these populations rather than being unique to any one family, and several rCNVs were abundant in both tribes. To test familial clustering, we compared the average coefficient of relationship of rCNV carriers to the overall coefficients of relationship of the tribes (**Figures S1-S2**). Consistently with these being common rCNVs persistent for many generations, the coefficients of relationship of carriers of the same rCNV did not differ from the overall coefficients of relationship in the tribes. In our observation, the rCNVs were transmitted in families in Mendelian fashion, but kindreds transmitting the rCNVs were distributed throughout the populations and as noted, even in both.

This study was not structured to identify *de novo* CNVs or to capture their effects. For such studies, parent-child trios are ideal. However, *de novo* CNVs and CNVs particular to only one family are well known, with *de novo* mutations accounting for as much as 25% of the CNV burden associated with autism, contributing to approximately one third of all autism cases, and a half to two thirds of cases arising in low-risk families (37). In Schizophrenia, *de novo* CNVs also play an important role (38), the genes disrupted being enriched for development and synaptic function. Relative to genes implicated in Schizophrenia via GWAS, these CNVs confer a high level of risk for schizophrenia, with ORs ranging for 3–30. Thus, they should be subject to strong negative selection (2, 38, 39) due to their contributions to Schizophrenia, and as pleiotropic risk factors in Cognitive disability, Epilepsy and Autism as well, all being phenotypes that can directly or indirectly lead to decreased fertility (38). Thus, CNVs associated with diseases causing decreased reproductive fitness must be constantly replenished in populations by *de novo* mutation.

Correspondingly, as a group, *de novo* CNVs are more likely to be deleterious than CNVs transmitted through several generations, or that persist in populations for hundreds of generations. However, any CNV leading to haploinsufficiency or excess of expression of a gene is likely to have downstream phenotypic consequences. The absence of rCNVs from cosmopolitan populations cannot be explained by neutral drift since the larger effective breeding size of these populations would support the maintenance of neutral variation. This suggests that the rCNVs identified in the Native American populations will have phenotypic significance, however any effects on ORs for substance use disorders and psychiatric diseases are small. Using transcriptomic analysis, we found extensive gene network changes representing *trans* effects on expression of other genes in carriers of the 6p21.33 rCNV. These effects are consistent with the evolutionary conservation of genes within the rCNV and the observation of phenotypic consequences or even lethality in mice in which the genes contained within this rCNV are deleted, further reinforcing the idea that rCNVs deleting or duplicating genes are likely to alter phenotype, and fitness even if they do not have catastrophic effects. However, an important limitation of our study was lack of a comprehensive set of physiological and biochemical measures that might have captured non-psychiatric or more subtle effects of rCNVs.

On an individual basis only one rCNV remained nearly significant for association to “any psychiatric disease”. This rCNV at 22q11.2 in the Velocardiofacial syndrome region, had an OR of 4.77 and an OR for AUD of 3.18, which, whilst approaching significance, did not survive correction for testing of multiple rCNVs. The ability to link a specific rCNV to AUD or other discrete disease phenotype is limited by sample size. The associations of the rCNV deletion of the Chr 22 VCF region to “Any psychiatric disorder” and AUD are therefore instructive since they are based on 27 rCNV carriers, 22 of whom had AUD, compared to the expected 16 based on the overall prevalence of AUD in this sample of PI. Furthermore, 24 out of the observed 27 heterozygotes had a psychiatric disorder. Thus, these ORs are far larger than for a typical GWAS finding in psychiatric disease – the risk effect being comparable to the effects of the functional *ALDH2* and *ADH1B* variants on AUD and alcohol drinking. This suggests that other rCNVs and CNVs might alter risk for AUD but with lower OR or lower abundances that would render their effects undetectable in samples of the size we studied. We limited our current analysis to rCNVs with frequencies > 2.2% such that a large effect on risk (OR = 10) could be detectable with 80% power, and via this approach we would also be able to detect effects with smaller ORs, albeit with reduced power.

The clinical manifestations of 22q11.21 deletions causing haploinsufficiency of 30–50 genes are diverse, ranging from cardiac anomalies, facial dysmorphism, developmental delay and schizophrenia. Collectively, 22q11.21 deletion CNVs are common, occurring in approximately 1:3000–1:4000 live births. However, smaller 22q11.21 duplications are even more common, representing 1:1600 live births in Denmark (40). These 22q11.21 duplications have been implicated in autism spectrum disorder, intellectual disability, and bipolar disorder, with the penetrance altered by the size of the duplicated segment and the genes duplicated (41). Together with variations in genetic background and environmental exposure, it is understandable that the structural diversity of 22q11.21 CNVs leads to phenotypic differences from individual to individual and family to family, and this can be taken as an observation likely to apply at least in part to other CNVs that duplicate or delete genes in a variable fashion.

The 22q11.21 rCNV associated with “Any psychiatric disorder” in this study duplicates genes plausibly altering brain function: *USP18, DGCR6*, and *PRODH. USP18* (Ubiquitin Specific Peptidase 18), a deubiquitinase, plays a critical regulatory role in immune response, particularly type I interferon (IFN-I) signaling. Dysregulation of USP18 has been implicated in a spectrum of diseases, including autoimmune disorders, cancer, and neurological conditions. *DGCR6* (DiGeorge Syndrome Critical Region Gene 6), exerts a regulatory role on neural crest cell migration and early neurodevelopment. *DGCR6* apparently modulates the expression of neighboring genes such as *TBX1* (T-Box Transcription Factor 1). *DGCR6* variants have been associated with schizophrenia susceptibility and brain connectivity, suggesting that duplications of this gene might have downstream effects on behavioral regulation. *PRODH* (Proline Dehydrogenase 1) encodes a key enzyme crucial for proline catabolism that impacts glutamatergic neurotransmission. Whilst *PRODH* genetic variants have been linked to impulsivity, cognitive deficits, and altered prefrontal cortex activity, increased gene dosage due to *PRODH* duplications perturbs proline-to-glutamate conversion, potentially disrupting the excitatory/inhibitory balance in key brain regions implicated in addiction vulnerability.

As previously mentioned, rCNVs may exert stronger effects on non-psychiatric phenotypes not analyzed in this study. The 20p12.1 rCNV found in both Native American populations deletes *MACROD2*, a deacetylase that specifically targets the removal of ADP-ribose from mono-ADP-ribosylated proteins. Mutations or dysregulation of *MACROD2* have been associated with several diseases, including neurodevelopmental disorders such as ASD and ID (42). Notably, the *MACROD2* rCNV had an OR of 4.9 for AUD (95% CI:1.1–22.1), potentially indicating a large effect.

The presence of rCNVs in Native Americans highlights the importance of expanding genomic studies to diverse populations. Native Americans and other well-defined populations carry alleles contributing to their unique characteristics and frequency of private alleles: an *HTR2B* stop codon that is rare worldwide has a frequency of 1.5% in the Finnish population (43) and a different *HTR2B* stop codon of somewhat lower abundance is exclusive to South Asians (Genome Aggregation Database (gnomAD)). Whilst many population-specific variants are thought to be non-pathogenic, some functional polymorphisms are maintained in particular populations by selection for diverse phenotypes and for example resistance to malaria (44) dietary lactose, (45) and more speculatively, plague (46).

It is unknown what proportion of heritability of AUD and other psychiatric phenotypes that ongoing expansion in size of GWAS will eventually capture, GWAS being performed with common SNPs either directly genotyped or imputed (47). Notably, certain CNVs in Schizophrenia such as 22q11.2 and 3q29 deletion, confer substantial individual risk with ORs ranging from 20–40. Other loci such as 16p11.2 duplications, 1q21.1 deletions, and *NRXN1* deletions exhibit more moderate effects with ORs ranging from 5 to 12 (2, 13, 48, 49). In contrast, the genetic liability conferred by rare CNVs in ASD has been estimated to account for 5–10% of cases, particularly through *de novo* events (50, 51). Other sources of genetic variance that are largely untapped by GWAS are rare SNVs and STR (short tandem repeat) polymorphisms whose genotypes are not readily captured by proxy SNPs. Prospectively, study of these additional types of genetic variation, and in contexts such as founder populations, may lead to the discovery of additional loci of large effect.

## Conclusions

The genetic risk for AUD and related psychiatric disorders arises from a complex interplay of common and rare variants, much of which remains unexplored. The enrichment of rCNVs in founder populations such as Native Americans suggests that CNVs can thereby be linked to psychiatric diagnoses in a way that is not possible in other populations. Our analysis, and others, show the *cis* and *trans* molecular consequences of CNVs that delete and duplicate genes. This has important implications for future studies in large and ancestrally diverse datasets where it will be possible to aggregate non-recurrent CNVs to deepen our understanding of psychiatric disease etiology. By moving beyond traditional GWAS to incorporate structural variants, future studies can uncover biological mechanisms that have so far remained hidden.

## Methods

### Subjects

We studied 395 members of a PI tribe in Oklahoma, and 370 members of a SWI tribe in Arizona, the names of the tribes being withheld. In both, participants were collected as part of AUD research involving super pedigrees selected based on structure and accessibility rather than relationship to an affected proband. In these Native American communities, AUD is common, and thus it was unnecessary to select pedigrees based on phenotype or relationship to an affected proband, and as was not done. KING-robust kinship coefficients were computed in PLINK2 using the KING algorithm, after exclusion of SNPs with a missing rate > 0.1 and minor allele frequency < 0.01 (52). The coefficients of relationship of the study participants were equivalent to those of the overall populations of these two tribes, and just below the second cousin level, as previously reported. The histograms of all pairwise relationships of the participants are shown (**Figure S1**). For comparison, we studied 1,458 unrelated individuals representing a cosmopolitan case/control AUD sample at the NIH CC, these patients also having high frequencies of other SUDs and other psychiatric disorders.

All Native American participants underwent assessment using the Structured Assessment for Diagnosis–Lifetime Version (SADS-LA) and the Structured Clinical Interview for DSM (SCID) was used for the NIH CC sample. Psychiatric diagnoses were assigned by consensus conference and via DSMIII-R criteria (PI and SWI) or DSM-5 criteria (NIH CC). All participants were studied under NIH IRB-approved human research protocols and provided informed consent in accordance with the Declaration of Helsinki, including consent for genomic studies, and as approved by the Tribal Councils of both tribes, thus also representing group consent.

### Genotyping

Genomic DNA prepared from LCLs (Native Americans) or venous blood (NIH CC sample). Two Illumina genotyping arrays were used: for PI, the Infinium HumanHap 550 array, which includes 561,466 SNPs of which 98,656 are located in regions of known CNVs; and for SWI and NIH CC, the Infinium Human OmniExpress Exome array, which includes 962,215 SNPs, of which 206,665 are located in regions of known CNVs (53). Samples with an overall call rate of < 98% were excluded. This resulted in the exclusion of 48 samples such that the final sample sizes were PI: 387, SWI: 350 and NIH CC: 1438. The genotype reproducibility rate was 0.999 based on 91 duplicate sample pairs.

For CNV calling, Log R Ratio (LRR) and B Allele Frequency (BAF) data were exported from normalized Illumina datasets using the CNV Partition tool in Genome Studio (Illumina). CNV detection was also performed using PennCNV (version 1.0.3), which applies a Hidden Markov Model (HMM) to integrate LRR, BAF, and SNP allele frequencies.

Only autosomal SNPs with known positions were considered and CNVs were retained only if detected by both CNV Partition and PennCNV, and if they spanned at least ten adjacent SNPs and were ≥ 10 kb in size. All CNV calls were manually reviewed by inspecting LRR and BAF plots. CNV annotation was performed using the hg19 (GRCh37) reference genome. rCNVs were initially identified based on common breakpoints and then confirmed as rCNVs by haplotype analysis, all candidate rCNVs being confirmed to be on the same haplotype backgrounds in the 3’ and 5’ regions immediate to the candidate rCNVs.

### Haplotype and Coalescence Analyses

To evaluate haplotype background, genotypes of SNPs flanking candidate rCNVs were examined utilizing Haploview 4.2 (54). The number of generations since each rCNV was introduced into the population by mutation or introgression was estimated using the Gamma method with an assumption of independence (55). The coalescence time of chromosomes with and without the allele was inferred based on the assumption that the shared haplotype of a variant, representing the distance to the first recombination site, decreases with an increasing number of generations. Among individuals who were not first- or second-degree relatives, 60 SNPs were selected both upstream and downstream of each rCNV and phased using Beagle 5.4 (56, 57). Shared haplotypes were successively extended via SNPs yielding congruent haplotypes or if the alleles at the five successive SNPs matched, thus “rescuing” point mutations and the rare genotyping error. The parameter τ was estimated by 2/ *l*_*ave*_, where *l*_*ave*_ is the weighted average of maximum shared haplotype sizes. The weights were derived from the total numbers of haplotypes that had at least one common breakpoint defining a maximum shared haplotype region. The sex-averaged local recombination rates were used, replacing the uniform recombination rate assumed in the Haldane model (58).

### Differential Expression Analysis

To detect *cis* and *trans* effects of a common rCNV on transcriptome, we compared gene expression in lymphoblastoid cell lines from five individuals with a 6p21.33 rCNV deletion against four individuals lacking the CNV, and we also evaluated *cis* effects of other CNV deletions and duplications in these nine LCL transcriptomes. Briefly, ~ 10 μg total RNA was reverse transcribed using the Superscript Vilo cDNA Synthesis Kit (Thermo Fisher Scientific, Waltham, MA) and the cDNA used for the Ion AmpliSeq^™^ Transcriptome Human Gene Expression Core Panel Kit (Thermo Fisher Scientific, Waltham, MA). Sequencing was performed using Ion 550 chips on an Ion S5 Sequencer (Thermo Fisher Scientific, Waltham, MA) yielding approximately 20 million reads per sample, with an average length of 115 bp. Reads were mapped to the hg19 genome reference in Torrent Suite^™^ Software 5.18.2. The differential gene expression was analyzed in R using DESeq2. Ingenuity Pathway Analysis (IPA) was used to identify canonical pathways and gene networks.

Associations of individual rCNVs observed in sufficient numbers for power to detect an effect of large size (see results) were evaluated by c2 test with Bonferroni adjustment, all expected cell sizes being > 5. Due to the numbers of rCNV carriers, the primary phenotypes tested were AUD versus no AUD and “no psychiatric diagnosis” versus “any psychiatric diagnosis” (including AUD, Phobia, MDD, PTSD, Obsessive Compulsive Disorder (OCD), SUD, Antisocial Personality Disorder (ASPD), Generalized Anxiety Disorder, Schizophrenia, and Bipolar Disorder).

### Burden Analyses

CNV burden was measured in two ways: number of CNVs and number of genes deleted or duplicated by CNVs. CNV burden was compared between cases and controls nonparametrically (Mann-Whitney Wilcoxon) for AUD and “any psychiatric disease”.

## Supplementary Material

Supplementary Files

This is a list of supplementary files associated with this preprint. Click to download.

- supplementary.docx

## Figures and Tables

**Table 1:** Demographic characteristics of Plains Indians, Southwest Indians and NIH CC samples.

Populations	Plains Indians	Southwest Indians	NIH CC
N	387	350	1438
Age (SD)	42.0 (14)	35.9 (13.1)	40.6 (13.3)
Sex (%)	169 (44)	150 (43)	851 (59)
Male	218 (56)	200 (57)	587 (41)
Female			

**Table 2:** Common Recurrent and Non-recurrent CNVs detected in Plains Indians and Southwest Indians.

Cytogenetic region	CNV type	Start (bp)	End (bp)	Size (Mb)	Carrier frequencies		Genes
					PI	SWI	
13q31.1	Deletion	84102440	84157927	0.05	80 (0.20)	-	*Non-genic region*
		84101480	84157927	0.05	-	51 (0.14)	
6p21.33	Deletion	31355318	31451476	0.09	60 (0.15)	-	*MICA, HCG-26 HCP-5, PMSP*
		31355318	31453640	0.09	-	93 (0.26)	
3p25.2	Duplication	12610706	12792622	0.18	43 (0.11)	-	*RAF1, TMEM40*
		31355318	31453640	0.09	-	93 (0.26)	
22q11.23-q12.1	Duplication	25661725	25910667	0.24	25 (0.06)	-	*LRP5L, CRYBB2P1*
		25650406	25910667	0.26	-	79 (0.22)	
20p12.1	Deletion	14758111	14830453	0.07	18 (0.04)	-	*MACROD2*
		14815778	15171838	0.35	-	2 (0.005)	
9p24.3	Duplication	526772	704075	0.17	7 (0.01)	-	*KANK1*
		483018	489338	0.006	-	2 (0.005)	
17p11.2-p11.1	Duplication	21704418	22242355	0.53	19 (0.04)	-	*MTRNR2L1*
		21539613	22242355	0.70	-	18 (0.05)	
12p13.31	Duplication	7996890	8123306	0.12	4 (0.01)	-	*SLC2A14, NANOGP1, SLC2A3*
		8000912	8114429	0.11	-	2 (0.005)	
7q31.1	Deletion	111085618	111242161	0.15	6 (0.01)	-	*IMMP2L*
		110971919	111309224	0.33	-	7 (0.02)	
19q13.42	Deletion	53932295	54014178	0.08	2 (0.005)	-	*ZNF761, ZNF813*
		53932295	54011384	0.07	-	3 (0.008)	
13q33.2	Deletion	105918084	106007135	0.08	2 (0.005)	-	*Non-genic region*
		106219874	106307349	0.08	-	20 (0.05)	
4q12	Duplication	58604281	58758826	0.15	1 (0.002)	-	*Non-genic region*
		58604281	58770663	0.16	-	7 (0.02)	
7q11.23	Duplication	76066189	76557212	0.49	1 (0.002)	-	*MULTIGENIC*
		76131646	76639871	0.50	-	6 (0.01)	
2q21.1	Deletion	132077379	132311088	0.23	1 (0.002)	-	*MULTIGENIC*
		132057166	132298468	0.24	-	5 (0.01)	
12q14.2	Duplication	63947694	64129108	0.18	2 (0.005)	-	*DPY19L2*
		63946056	64118558	0.17	-	2 (0.005)	
1p13.3	Deletion	109705022	109832283	0.12	2 (0.005)	-	*CELSR2*
		109789795	109820919	0.03	-	5 (0.01)	

**Table 3:** rCNV associations with AUD and “Any Psychiatric Disorders” in Plains Indians (PI) and Southwest Indians (SWI).

CNV region	Dataset Presence	AUD			Any Psychiatric Disorder			No. of carriers
		OR	CI (95%)	p-value	OR	CI (95%)	p-value	
6p21.33_del	PI & SWI	1.16	0.91–1.49	0.43	1.17	0.91–1.51	0.45	153
13q31.1_del	PI & SWI	1.13	0.86–1.49	0.55	0.99	0.75–1.31	0.96	127
22q11.23_dup	PI & SWI	0.83	0.60–1.14	0.40	1.05	0.76–1.45	0.84	97
11q11_del	PI	1.10	0.67–1.81	0.72	1.03	0.62–1.72	0.91	83
1q21.3_del	PI	1.19	0.68–2.06	0.55	1.29	0.73–2.29	0.38	65
3p25.2_dup	PI	0.89	0.46–1.70	0.72	0.88	0.45–1.71	0.71	29
9p23_dup	PI	1.11	0.51–2.43	0.79	1.05	0.47–2.32	0.91	27
19q13.42_del	PI	1.75	0.75–4.09	0.09	2.01	0.79–5.11	0.13	27
22q11.21_dup	PI	3.18	1.18–8.59	**0.01**	4.77	1.41–16.15	**0.006**	27
7p22.2_del	PI	2.02	0.83–4.90	0.11	1.62	0.67–3.94	0.28	26
22q11.22_del	PI	0.47	0.21–1.05	0.06	0.87	0.38–4.63	0.74	26
8p23.2_del	PI	1.80	0.73–4.42	0.19	1.80	0.70–4.63	0.21	25
6q14.1_del	PI	1.21	0.52–2.82	0.65	1.18	0.50–2.81	0.70	25
7p22.1_del	SWI	1.68	0.80–3.53	0.17	0.96	0.42–2.19	0.93	47
7p22.3_dup	SWI	1.12	0.52–2.42	0.77	0.99	0.39–2.51	0.99	36
2q37.3_del	SWI	2.12	0.85–5.29	0.10	1.55	0.52–4.59	0.43	34
16p13.3_del	SWI	1.31	0.57–3.02	0.53	1.08	0.40–2.94	0.88	32
8q21.3_del	SWI	0.88	0.40–1.95	0.75	0.53	0.22–1.26	0.15	31
3q26.31_dup	SWI	0.84	0.38–1.86	0.66	0.51	0.21–1.21	0.12	30
4p16.3_del	SWI	1.22	0.47–3.19	0.69	0.53	0.20–1.42	0.20	23
6p21.33_del	SWI	0.97	0.39–2.43	0.95	0.69	0.25–1.96	0.49	23
7p36.1_del	SWI	1.57	0.57–4.37	0.38	0.94	0.31–2.88	0.91	23
13q12.11_dup	SWI	0.73	0.29–1.79	0.48	0.89	0.29–2.73	0.83	22
17q12_dup	SWI	0.59	0.24–1.43	0.24	1.28	0.36–4.47	0.70	22
19q13.41_dup	SWI	0.59	0.24–1.43	0.24	0.50	0.19–1.34	0.16	22

Bolded results are statistically significant (p< 0.05)

## Data Availability

The data supporting the analyses and findings of this study are available on request from the corresponding author.

## Abbreviations

AUD: Alcohol Use Disorder
ASPD: Antisocial Personality Disorder
BAF: B Allele Frequency
CNVs: Copy Number Variants
GSTM1: Glutathione s-transferase
HMM: Hidden Markov Model
IPA: Ingenuity Pathway Analysis
ID: Intellectual disability
LRR: Log R Ratio
LCLs: Lymphoblastoid cell lines
MDD: Major Depressive Disorder
NIH CC: NIH Clinical Center
NAHR: Nonallelic homologous recombination
OCD: Obsessive Compulsive Disorder
OR: odds ratio
PI: Plains Indians
PTSD: Post-Traumatic Stress Disorder
rCNV: Recurrent CNV
SNPs: Single nucleotide polymorphisms
SWI: Southwest Indians
SADS-LA: Structured Assessment for Diagnosis–Lifetime Version
SCID: Structured Clinical Interview for DSM
SUDs: Substance Use Disorders
VCF: Velocardiofacial Syndrome

## References

[1] Zarrei M, Burton CL, Engchuan W, Young EJ, Higginbotham EJ, MacDonald JR (2019) A large data resource of genomic copy number variation across neurodevelopmental disorders. NPJ Genom Med 4:26 31602316 10.1038/s41525-019-0098-3 PMC6779875

[2] Marshall CR, Howrigan DP, Merico D, Thiruvahindrapuram B, Wu W, Greer DS (2017) Contribution of copy number variants to schizophrenia from a genome-wide study of 41,321 subjects. Nat Genet 49(1):27–35 27869829 10.1038/ng.3725 PMC5737772

[3] Cook EH Jr., Scherer SW (2008) Copy-number variations associated with neuropsychiatric conditions. Nature 455(7215):919–923 18923514 10.1038/nature07458

[4] McDonald-McGinn DM, Sullivan KE, Marino B, Philip N, Swillen A, Vorstman JA (2015) 22q11.2 deletion syndrome. Nat Rev Dis Primers 1:15071 27189754 10.1038/nrdp.2015.71 PMC4900471

[5] Yobb TM, Somerville MJ, Willatt L, Firth HV, Harrison K, MacKenzie J (2005) Microduplication and triplication of 22q11.2: a highly variable syndrome. Am J Hum Genet 76(5):865–876 15800846 10.1086/429841 PMC1199375

[6] Bray NJ, O’Donovan MC (2019) The genetics of neuropsychiatric disorders. Brain Neurosci Adv.;2 10.1177/2398212818799271 PMC6551216 31179400

[7] Dilliott AA, Zhang KK, Wang J, Abrahao A, Binns MA, Black SE (2022) Targeted copy number variant identification across the neurodegenerative disease spectrum. Mol Genet Genomic Med 10(8):e1986 35666053 10.1002/mgg3.1986 PMC9356547

[8] Girirajan S, Campbell CD, Eichler EE (2011) Human copy number variation and complex genetic disease. Annu Rev Genet 45:203–226 21854229 10.1146/annurev-genet-102209-163544 PMC6662611

[9] Yuen RKC, Merico D, Bookman M, Thiruvahindrapuram JLH, Patel B (2017) Whole genome sequencing resource identifies 18 new candidate genes for autism spectrum disorder. Nat Neurosci 20(4):602–611 28263302 10.1038/nn.4524 PMC5501701

[10] Stankiewicz P, Lupski JR (2010) Structural variation in the human genome and its role in disease. Annu Rev Med 61:437–455 20059347 10.1146/annurev-med-100708-204735

[11] Cardoso AR, Oliveira M, Amorim A, Azevedo L (2016) Major influence of repetitive elements on disease-associated copy number variants (CNVs). Hum Genomics 10(1):30 27663310 10.1186/s40246-016-0088-9 PMC5035501

[12] McCarroll SA (2010) Copy number variation and human genome maps. Nat Genet 42(5):365–366 20428091 10.1038/ng0510-365

[13] Rees E, Walters JT, Georgieva L, Isles AR, Chambert KD, Richards AL (2014) Analysis of copy number variations at 15 schizophrenia-associated loci. Br J Psychiatry 204(2):108–114 24311552 10.1192/bjp.bp.113.131052 PMC3909838

[14] Kirov G, Rees E, Walters JT, Escott-Price V, Georgieva L, Richards AL (2014) The penetrance of copy number variations for schizophrenia and developmental delay. Biol Psychiatry 75(5):378–385 23992924 10.1016/j.biopsych.2013.07.022 PMC4229045

[15] Hu Z, Xiao X, Zhang Z, Li M (2019) Genetic insights and neurobiological implications from NRXN1 in neuropsychiatric disorders. Mol Psychiatry 24(10):1400–1414 31138894 10.1038/s41380-019-0438-9

[16] Rees E, Kirov G (2021) Copy number variation and neuropsychiatric illness. Curr Opin Genet Dev 68:57–63 33752146 10.1016/j.gde.2021.02.014 PMC8219524

[17] Cooper GM, Coe BP, Girirajan S, Rosenfeld JA, Vu TH, Baker C (2011) A copy number variation morbidity map of developmental delay. Nat Genet 43(9):838–846 21841781 10.1038/ng.909 PMC3171215

[18] Pinto D, Delaby E, Merico D, Barbosa M, Merikangas A, Klei L (2014) Convergence of genes and cellular pathways dysregulated in autism spectrum disorders. Am J Hum Genet 94(5):677–694 24768552 10.1016/j.ajhg.2014.03.018 PMC4067558

[19] Malwade S, Ingason A, Khodosevich K (2025) The use of single-cell and spatial omics to study copy number variants. Biol Psychiatry10.1016/j.biopsych.2025.06.010 40545020

[20] Zhang X, Abdellaoui A, Rucker J, de Jong S, Potash JB, Weissman MM (2019) Genome-wide Burden of Rare Short Deletions Is Enriched in Major Depressive Disorder in Four Cohorts. Biol Psychiatry 85(12):1065–1073 31003785 10.1016/j.biopsych.2019.02.022 PMC6750266

[21] Kendall KM, Rees E, Bracher-Smith M, Legge S, Riglin L, Zammit S (2019) Association of Rare Copy Number Variants With Risk of Depression. JAMA Psychiatry 76(8):818–825 30994872 10.1001/jamapsychiatry.2019.0566 PMC6583866

[22] Antshel KM, Zhang-James Y, Wagner KE, Ledesma A, Faraone SV (2016) An update on the comorbidity of ADHD and ASD: a focus on clinical management. Expert Rev Neurother 16(3):279–293 26807870 10.1586/14737175.2016.1146591

[23] Coe BP, Witherspoon K, Rosenfeld JA, van Bon BW, Vulto-van Silfhout AT, Bosco P (2014) Refining analyses of copy number variation identifies specific genes associated with developmental delay. Nat Genet 46(10):1063–1071 25217958 10.1038/ng.3092 PMC4177294

[24] Kendall KM, Rees E, Escott-Price V, Einon M, Thomas R, Hewitt J (2017) Cognitive Performance Among Carriers of Pathogenic Copy Number Variants: Analysis of 152,000 UK Biobank Subjects. Biol Psychiatry 82(2):103–110 27773354 10.1016/j.biopsych.2016.08.014

[25] Reale L, Bartoli B, Cartabia M, Zanetti M, Costantino MA, Canevini MP (2017) Comorbidity prevalence and treatment outcome in children and adolescents with ADHD. Eur Child Adolesc Psychiatry 26(12):1443–1457 28527021 10.1007/s00787-017-1005-z

[26] Martin J, Tammimies K, Karlsson R, Lu Y, Larsson H, Lichtenstein P, Magnusson PKE (2019) Copy number variation and neuropsychiatric problems in females and males in the general population. Am J Med Genet B Neuropsychiatr Genet 180(6):341–350 30307693 10.1002/ajmg.b.32685 PMC6767107

[27] Martin J, Hosking G, Wadon M, Agha SS, Langley K, Rees E (2020) A brief report: de novo copy number variants in children with attention deficit hyperactivity disorder. Transl Psychiatry 10(1):135 32398668 10.1038/s41398-020-0821-y PMC7217839

[28] Dawson DA, Goldstein RB, Saha TD, Grant BF (2015) Changes in alcohol consumption: United States, 2001–2002 to 2012–2013. Drug Alcohol Depend 148:56–61 25620731 10.1016/j.drugalcdep.2014.12.016 PMC4330106

[29] Kwako LE, Momenan R, Litten RZ, Koob GF, Goldman D (2016) Addictions Neuroclinical Assessment: A Neuroscience-Based Framework for Addictive Disorders. Biol Psychiatry 80(3):179–189 26772405 10.1016/j.biopsych.2015.10.024 PMC4870153

[30] Zhang H, Grant BF, Hodgkinson CA, Ruan WJ, Kerridge BT, Huang B (2022) Strong and weak cross-inheritance of substance use disorders in a nationally representative sample. Mol Psychiatry 27(3):1742–1753 34759357 10.1038/s41380-021-01370-0 PMC9085976

[31] Zhou H, Gelernter J (2024) Human genetics and epigenetics of alcohol use disorder. J Clin Invest.;134(16) 10.1172/JCI172885 PMC11324314 39145449

[32] Ulloa AE, Chen J, Vergara VM, Calhoun V, Liu J (2014) Association between copy number variation losses and alcohol dependence across African American and European American ethnic groups. Alcohol Clin Exp Res 38(5):1266–1274 24512105 10.1111/acer.12364 PMC3999255

[33] Torres EM, Springer M, Amon A (2016) No current evidence for widespread dosage compensation in S. cerevisiae. Elife 5:e10996 26949255 10.7554/eLife.10996 PMC4798953

[34] Hayes JD, Strange RC (2000) Glutathione S-transferase polymorphisms and their biological consequences. Pharmacology 61(3):154–166 10971201 10.1159/000028396

[35] Urbanek M, Goldman D, Long JC (1996) The apportionment of dinucleotide repeat diversity in Native Americans and Europeans: a new approach to measuring gene identity reveals asymmetric patterns of divergence. Mol Biol Evol 13(7):943–953 8752003 10.1093/oxfordjournals.molbev.a025662

[36] Peltonen L, Palotie A, Lange K (2000) Use of population isolates for mapping complex traits. Nat Rev Genet 1(3):182–190 11252747 10.1038/35042049

[37] Yoon S, Munoz A, Yamrom B, Lee YH, Andrews P, Marks S (2021) Rates of contributory de novo mutation in high and low-risk autism families. Commun Biol 4(1):1026 34471188 10.1038/s42003-021-02533-z PMC8410909

[38] Rees E, Kirov G, O’Donovan MC, Owen MJ (2012) De novo mutation in schizophrenia. Schizophr Bull 38(3):377–381 22451492 10.1093/schbul/sbs047 PMC3329988

[39] Gulsuner S, Walsh T, Watts AC, Lee MK, Thornton AM, Casadei S (2013) Spatial and temporal mapping of de novo mutations in schizophrenia to a fetal prefrontal cortical network. Cell 154(3):518–529 23911319 10.1016/j.cell.2013.06.049 PMC3894107

[40] Olsen L, Sparso T, Weinsheimer SM, Dos Santos MBQ, Mazin W, Rosengren A (2018) Prevalence of rearrangements in the 22q11.2 region and population-based risk of neuropsychiatric and developmental disorders in a Danish population: a case-cohort study. Lancet Psychiatry 5(7):573–580 29886042 10.1016/S2215-0366(18)30168-8 PMC6560180

[41] Hoeffding LK, Trabjerg BB, Olsen L, Mazin W, Sparso T, Vangkilde A (2017) Risk of Psychiatric Disorders Among Individuals With the 22q11.2 Deletion or Duplication: A Danish Nationwide, Register-Based Study. JAMA Psychiatry 74(3):282–290 28114601 10.1001/jamapsychiatry.2016.3939

[42] Jones RM, Cadby G, Blangero J, Abraham LJ, Whitehouse AJO, Moses EK (2014) MACROD2 gene associated with autistic-like traits in a general population sample. Psychiatr Genet 24(6):241–248 25360606 10.1097/YPG.0000000000000052 PMC4320645

[43] Bevilacqua L, Doly S, Kaprio J, Yuan Q, Tikkanen R, Paunio T (2010) A population-specific HTR2B stop codon predisposes to severe impulsivity. Nature 468(7327):1061–1066 21179162 10.1038/nature09629 PMC3183507

[44] Driss A, Hibbert JM, Wilson NO, Iqbal SA, Adamkiewicz TV, Stiles JK (2011) Genetic polymorphisms linked to susceptibility to malaria. Malar J 10:271 21929748 10.1186/1475-2875-10-271 PMC3184115

[45] Anguita-Ruiz A, Aguilera CM, Gil A (2020) Genetics of Lactose Intolerance: An Updated Review and Online Interactive World Maps of Phenotype and Genotype Frequencies. Nutrients.;12(9) 10.3390/nu12092689 PMC7551416 32899182

[46] Stephens JC, Reich DE, Goldstein DB, Shin HD, Smith MW, Carrington M (1998) Dating the origin of the CCR5-Delta32 AIDS-resistance allele by the coalescence of haplotypes. Am J Hum Genet 62(6):1507–1515 9585595 10.1086/301867 PMC1377146

[47] Sullivan PF, Daly MJ, O’Donovan M (2012) Genetic architectures of psychiatric disorders: the emerging picture and its implications. Nat Rev Genet 13(8):537–551 22777127 10.1038/nrg3240 PMC4110909

[48] Mulle JG, Dodd AF, McGrath JA, Wolyniec PS, Mitchell AA, Shetty AC (2010) Microdeletions of 3q29 confer high risk for schizophrenia. Am J Hum Genet 87(2):229–236 20691406 10.1016/j.ajhg.2010.07.013 PMC2917706

[49] Mollon J, Almasy L, Jacquemont S, Glahn DC (2023) The contribution of copy number variants to psychiatric symptoms and cognitive ability. Mol Psychiatry 28(4):1480–1493 36737482 10.1038/s41380-023-01978-4 PMC10213133

[50] Pinto D, Pagnamenta AT, Klei L, Anney R, Merico D, Regan R (2010) Functional impact of global rare copy number variation in autism spectrum disorders. Nature 466(7304):368–372 20531469 10.1038/nature09146 PMC3021798

[51] Sanders SJ, He X, Willsey AJ, Ercan-Sencicek AG, Samocha KE, Cicek AE (2015) Insights into Autism Spectrum Disorder Genomic Architecture and Biology from 71 Risk Loci. Neuron 87(6):1215–1233 26402605 10.1016/j.neuron.2015.09.016 PMC4624267

[52] Manichaikul A, Mychaleckyj JC, Rich SS, Daly K, Sale M, Chen WM (2010) Robust relationship inference in genome-wide association studies. Bioinformatics 26(22):2867–2873 20926424 10.1093/bioinformatics/btq559 PMC3025716

[53] Marenne G, Rodriguez-Santiago B, Closas MG, Perez-Jurado L, Rothman N, Rico D (2011) Assessment of copy number variation using the Illumina Infinium 1M SNP-array: a comparison of methodological approaches in the Spanish Bladder Cancer/EPICURO study. Hum Mutat 32(2):240–248 21089066 10.1002/humu.21398 PMC3230937

[54] Barrett JC, Fry B, Maller J, Daly MJ (2005) Haploview: analysis and visualization of LD and haplotype maps. Bioinformatics 21(2):263–265 15297300 10.1093/bioinformatics/bth457

[55] Gandolfo LC, Bahlo M, Speed TP (2014) Dating rare mutations from small samples with dense marker data. Genetics 197(4):1315–1327 24879464 10.1534/genetics.114.164616 PMC4125402

[56] Browning BL, Zhou Y, Browning SR (2018) A One-Penny Imputed Genome from Next-Generation Reference Panels. Am J Hum Genet 103(3):338–348 30100085 10.1016/j.ajhg.2018.07.015 PMC6128308

[57] Browning BL, Tian X, Zhou Y, Browning SR (2021) Fast two-stage phasing of large-scale sequence data. Am J Hum Genet 108(10):1880–1890 34478634 10.1016/j.ajhg.2021.08.005 PMC8551421

[58] Kong A, Gudbjartsson DF, Sainz J, Jonsdottir GM, Gudjonsson SA, Richardsson B (2002) A high-resolution recombination map of the human genome. Nat Genet 31(3):241–247 12053178 10.1038/ng917

